# Mapping the Network of Social Cognition Domains in Children With Autism Spectrum Disorder Through Graph Analysis

**DOI:** 10.3389/fpsyt.2020.579339

**Published:** 2020-10-30

**Authors:** Maria Chiara Pino, Roberto Vagnetti, Francesco Masedu, Margherita Attanasio, Sergio Tiberti, Marco Valenti, Monica Mazza

**Affiliations:** ^1^Department of Applied Clinical Sciences and Biotechnology, University of L'Aquila, L'Aquila, Italy; ^2^Regional Centre for Autism, Abruzzo Region Health System, L'Aquila, Italy

**Keywords:** autism spectrum disorder, graph analysis, network, social cognition, verbal mental age

## Abstract

Children with autism spectrum disorder (ASD) are characterized by difficulties in social cognition (SC) domains. The aim of this study is to build an SC network to explore associations among interacting elements within this cognitive construct. We used a graph analysis to explain how individual SC domains relate to each other and how these relations may differ between ASD and typically developing (TD) groups. Seventy-six children with ASD and 81 TD children, matched for verbal mental age, were subjected to three SC measures. Our results showed that TD children exhibited an SC network characterized by a single domain (i.e., social cognition), while children with ASD demonstrated communicating node communities where social information processing measured by the Social Information Processing Interview (SIPI) represents a key point in understanding network differences between groups.

## Introduction

Social cognition (SC) is an adaptive human function (1, 2) that includes cognitive domains encompassing the capacity to process the social world (such as emotional processing, attention, social stimuli encoding, etc.). It emerges in early childhood through the development of “theory of mind” [ToM; (3)], that is, the capacity to understand mental and emotional states of other people (2). The development of SC competencies follows a certain sequential order in typically developing (TD) children (3, 4). Children with autism spectrum disorder (ASD) display the same progressive order, but this appears to be delayed in terms of age at attainment (3, 5). In conformance with this view, recent literature (3, 6) has suggested the ASD profile is best described in terms of a delay in the development of these social abilities, as opposed to a total lack of SC competencies. This view has important implications, bringing into focus the need for early rehabilitation programs geared toward improving social competencies, attenuating social deficits in children with autism, and reducing the child's isolation. In fact, prevalence of ASD in the general population is estimated worldwide to be around 1% both from screening and register-based studies (7). We know that SC domains do not operate in isolation, yet it remains undefined how difficulty in this complex social construct influences interactions among different social domains. The interrelationship among SC domains is crucial to developing social behaviors that are adequate to their surrounding context and to maintaining social relationships. According to Ibrahim et al. (8), one method of exploring associations among interacting elements within a complex system as SC involves their separation into networks that can be characterized and quantified through graphical theoretical analysis. The graph analysis explains how individual variables such as SC outcomes relate to each other, how these relations may differ between groups, and how information may be exchanged. The current study applies graph theory to social behavioral data in children with ASD in order to better understand which social domains are important in an interacting network compared to TD children.

In constructing the network, we consider that SC is a complex structure that could involve many domains, in this case, nodes that—according to Happé and Frith (9)—can be understood as a complex network diagram that includes distinct components. The main SC component that appears to be compromised in ASD concerns ToM. The ToM ability required a good capacity to understand emotions, and it can affect social information processing. TD children begin to consolidate ToM ability at the age of 4–5 years (3, 9). Around the age of 6 years, this ability starts to become more elaborate and allow the development of prosocial behavior. These abilities must communicate effectively with each other to allow their proper development. In individuals with ASD, delayed development of SC abilities could compromise the construction of effective networks with consequent impairment of all their social skills.

Our study aims to investigate a simplified model of Happé and Frith's (9) theoretical framework, based on observed data, in order to graphically represent their idea of SC network and relations among components. Moreover, graphical analysis allows the devising and testing of connections between components with associated path properties (8).

In order to obtain a more simplified network that could lead to a better understanding of interactions among elements, we focused our approach on underlying differences between ASD and TD, taking into consideration that ToM is the main core of ASD symptoms (10). In this study, we used three SC tests: (1) a simplified version of the Eyes Task [ET; (11)]; (2) the Comic Strip Task [CST; (12)], a well-known test assessing different components of ToM, i.e., intentions, emotions, and beliefs; and (3) the Social Information Processing Interview [SIPI; (13, 14)] to evaluate the capacity to process social information and display adequate social behavior. While the CST is based on seeing social scenes, the ET is focused on the understanding of others by their gaze. Even if two tests evaluated the same construct, they used a different kind of information and analysis for their contribution within the network; they could show whether they effectively work in the same way or if the kind of information is elaborated in a different manner. We decided to include the results of the SIPI in the network because it is based on Crick and Dodge's (15) model and can summarize many nodes of Happè and Frith's (9) map. In fact, the processes described by Crick and Dodge (15) are related to many dimensions, i.e., social schemas and rules, self and other evaluations, evaluation of goal and arousal regulation, that are used to determine SIPI scores.

A topographic view of the interaction of these domains in ASD could lead to a greater understanding of the associations between social deficits and ASD symptomatology, providing new opportunities for diagnosis and treatment. This study is the first to use graph theory measures to investigate the SC network in children with ASD. For this reason, we investigated the global SC network in children with ASD using TD children as a control.

## Methods

### Participants

The participant group in our study includes 76 children with ASD selected by the Reference Regional Center for Autism, Abruzzo Region Health System, L'Aquila, Italy [mean ± standard deviation chronological age (CA) = 9.21 ± 2.65 years; 70 males and six females] and 81 TD children recruited from a local primary school in the same region (mean ± SD CA = 6.39 ± 1.69 years; 70 males and 11 females) for a total of 157 children. Groups were matched by verbal mental age (VMA) through the Test for Reception of Grammar-Version 2 (TROG-2; 14; TD-VMA = 7.11 ± 2.09; ASD-VMA = 7.43 ± 2.72). Inclusion criteria for the ASD population were: (1) a diagnosis of ASD made by the Autism Diagnostic Observation Schedule-Second Version [ADOS-2; (16)] and (2) no other neurological disorders. Required for inclusion in the TD group was the absence of any neurological or psychological disorder. Demographic data of samples are reported in Table 1.

**Table 1:** Demographic data of the sample and clinical information concerning the ASD group.

	**TD group** **(*n* = 81)** **Mean (SD)**	**ASD group** **(*n* = 76)** **Mean (SD)**	***t*** **df(1, 155)**	***p***
**DEMOGRAPHIC DATA**				
Chronological age	6.39 (1.69)	9.21 (2.65)	−7.89	<0.01
Verbal mental age	7.11 (2.09)	7.43 (2.72)	−0.82	0.41
**CLINICAL INFORMATION**				
ADOS-social communication and social interaction	–	9.48 (2.93)	–	–
ADOS-repetitive and stereotyped behaviors	–	1.29 (1.2)	–	–
ADOS total scores	–	10.78 (3.11)	–	–

*ADOS, Autism Diagnostic Observation Schedule; ADS, autism spectrum disorder; TD, typically developing*.

### Criterion for Group Matching

In our study, the children with autism had a CA higher than TD children because we chose to match the two groups on VMA assessed with the TROG-2 (17). The matching leads to chronologically older ASD [*t*_(155)_ = −7.89, *p* < 0.01] and to homogeneous groups in relation to their VMA [*t*_(155)_ = −0.82, *p* = 0.41].

According to recent literature (3), children with ASD showed a delay in developing SC abilities based on CA, whereas VMA appears to be a good predictor of ToM abilities altogether (18) and in children with autism (3). Indeed, Pino et al. (3) showed that ToM scores on the VMA in the ASD group had a significant relationship between this variable and mental states (both positive and negative), as measured with the ET and the intentions component of the CST. In addition, Hobson (19) showed that children with ASD succeeded on ToM tasks as could be expected from their mental age; thus, it can be argued that ToM tests require a specific cognitive ability, which could correspond to VMA.

### Social Cognition Measures

#### Eyes Task-Simplified (11)

This measure contains 56 black-and-white photos of children's eye region, portraying either mental states or primary emotions. The expressions included as positive primary emotions (PPE-ET) were happy or surprised, sad and angry were used for negative primary emotions (NPE-ET), while excited and thinking represented positive mental state (PMS-ET), and worried and shy were used for negative mental states (NMS-ET)—for a total of four emotional states and four mental states. Each stimulus involved one image (children's eye region), one target word on the left and the other with a target word on the right edge of the slide. Participants were asked to indicate which word best described the picture presented (e.g., “Look at this child—Is this child happy or angry?”). Testing lasted ~15 min based on the randomized presentation of the items. Accuracy was measured on a 0–7 scale based on the participant's selection of one of two choices in seven comparisons per target emotion/mental state [for example: 1. happy vs. surprised; 2. happy vs. sad; 3. happy vs. angry; 4. happy vs. excited; 5. happy vs. thinking; 6. happy vs. worried; 7. happy vs. shy; for details, including an example of an image of the test, see (11)].

#### Comic Strip Task (20)

The CST is a recent 21-item measure developed to assess three aspects of ToM: beliefs (B-CST), intentions (I-CST), and emotions (E-CST). Each subscale includes five items, each comprising a five-picture comic strip illustrating everyday social scenarios involving interpersonal interactions familiar to young children, for example, a birthday party or play at the park. In addition, it contains a control subscale (two items for each subcomponent of ToM) comprising non-social scenarios, albeit in settings similar to the other comic strips. According to the procedure proposed by Sivaratnam et al. (12), prior to the administration of the CST, six images depicting basic emotions (happy, sad, angry, frightened, surprised, and confused) were presented to ensure that the child was able to recognize basic emotions at the level required by the task. Specifically, the child was required to name at least four out of six images correctly to proceed with the ToM test. The CST subscales (Intentions–Beliefs–Emotions) were administered in the same order in both groups. For each item, children are shown three pictures that tell a social story, after which they are presented with two pictures containing alternative endings to the story and asked to select the one they think best completes the story. One option indicates a lack of understanding of others' mental states and is scored 0; the other indicates the presence of such understanding and is scored 1. For example, as could be seen in Sivaratnam et al. (12), item 5, “Recognition surprise,” from the Emotions subscale, in the first three figures is represented the protagonist involved in a surprise birthday party, where he is given gifts; then the child must choose whether: (1) the protagonist will be angry and he will run away or (2) the protagonist will be happy and he will celebrate with his friends. Each ToM subscale has a maximum score of 5, with a total test score of 15, with higher scores indicating superior ToM ability [for details, including an example of an image of the test, see (12)].

#### Social Information Processing Interview (13, 14)

The SIPI is an interview based on a storybook easel depicting a series of vignettes in which a protagonist is either rejected by two other peers or provoked by another peer. Each type of vignette is combined with each type of peer intent to generate four stories: (1) a non-hostile peer entry rejection story, (2) an ambiguous peer entry rejection story, (3) an accidental provocation story, and (4) an ambiguous provocation story. According to Ziv et al. (14), the scores correspond to four of the five mental steps of social information processing proposed by Crick and Dodge's (15) model: (1) Encoding, (2) Interpretation of cues, (3) Response construction, and (4) Response evaluation.

An example of an SIPI story is the following: Michael is watching the other children playing. Michael walks up to the other children and asks them: “Can I play with you?” The children say: “Sorry. The teacher said only two can play in the block area” [for details, see (14)]. The encoding component evaluates the level of detail that the child recalls across the four stories. Thus, the examiner asks the child: “Tell me what happened in the story, from the beginning to the end.” A score of 0 is given to a child who recalls no correct details for each story, and a score of 1 is given to a child who correctly recalls all the details in each story, and then, a sum of the scores is calculated, so scores could range from 0 (no correct coding in four stories) to 4 (all correct coding in four stories). The interpretation component evaluates the hostile attribution to others' behavior (the question is “Do you think the other children who didn't let Michael play are mean or not mean?”). The range for this score is 0–1 for each story, with higher scores representing higher levels of hostile attribution bias. The score in the interpretation component is inversely encoded compared with the other SIPI components; that is, a higher score indicates a major tendency to consider the behavior of other children as hostile. Regarding the response construction component, as reported by Ziv and Sorongon [(13), p. 7] in their paper, the score is derived from the child's responses to the open-ended item, “What would you say or do if this happened to you? The answers are used to create three, mutually exclusive flag variables (coded 0, 1) for each story: competent flag, aggressive flag, and avoidant flag. For example, if the child's response is coded as “competent,” then he or she is given a “1” for the competent flag, a “0” for the aggressive flag, and a “0” for the avoidant flag. The final response construction score is then calculated by subtracting the aggressive and avoidant scores from the competent score. The original range of this score is −4 (only avoidant or aggressive responses) to 4 (only competent responses). However, to avoid negative scale scores, the scale was modified such that the presented possible range for this score is 0 (only avoidant or aggressive responses) to 8 (only competent responses)” [(12), p. 7; (21)].

For the response evaluation step, the scores are made from a combination of 36 Response Evaluation questions (4 stories × 3 competent/aggressive/avoidant presented responses × 3 questions per presented response).

An example of a question for the response evaluation of an SIPI story is “Michael could kick apart the blocks and say to the other children, ‘if I can't play, then you can't play either,' (1) ‘Is this a good thing or a bad thing for Michael to say?,' (2) ‘If you do that, do you think the other children would like you?,' and (3) ‘Do you think the other children would let you play if you do that?”' If a child evaluates as positive an aggressive behavior, the score is 0. For example, child could respond that Michael said a good thing and that other children would let Michael play with them after his aggressive behavior, in this case, the given score is 0 (21).

### Statistical Analysis

#### Comparison Between Groups

The *T*-test was used to test the differences between the groups (ASD, TD) regarding demographic parameters and the measures for the components of the ET (positive primary emotions, negative primary emotions, positive mental states, and negative mental states) and for response evaluation of the SIPI subscale. The Mann–Whitney test was used to test differences between ASD and TD regarding the CST subscale (emotions, beliefs, and intention) and for the SIPI subscale of encoding, interpretation, and response construction). Bonferroni's correction has been made (α = 0.05).

#### Network Analysis

Graphs give a better way of dealing with abstract concepts like relationships and interactions; they provide an intuitive way of thinking these concepts (22, 23). Ibrahim et al. (8) propose that graph theory concepts are useful in the analysis of complex networks. By using graph theory, we can visualize and analyze relationships between SC domains. In graph theory, variables are termed “nodes,” which are connected *via* “edges.” Edges can be weighted: an edge with a higher weight is more strongly connected with a node than an edge with a lower weight. Moreover, edges can be directed, meaning that the edge between nodes A and B is different from the edge between nodes B and A. In our study, the SC measures constitute the nodes of the network, with the partial correlations between them as weighted and undirected edges. In graph analysis, there are several properties that can be inferred from a network. Some of the canonical centrality indices are represented by strength, betweenness, and closeness (24).

#### Network Analysis: Construction of the Network

Two graphs were constructed, one for the ASD group and one for the TD group, while nodes were represented by psychological domains that represent SC components (SC measures previously explained). Networks were estimated according to the Gaussian graphical model (25), in which edges are interpreted as partial correlation coefficients. We estimated the Gaussian graphical model using graphical least absolute shrinkage and selection operator (LASSO) (26), which estimates the (inverse) partial covariance matrix of a set of variables, penalizing small covariances by setting small covariances to zero. The optimal strength of the penalty term was estimated using the BIC (27). As some variables are ordinals, polychoric, polyserial, and Pearson correlations were used; moreover, only significant correlations were maintained.

After obtaining one network for each group, we evaluated several canonical proprieties (24) such as *strength, betweenness*, and *closeness* of each node.

*Strength* is a weighted measure of degree between a node and any other node connected to it, given by the formula:

$$\begin{array}{l} k_{i}=\underset{j\in N}{\sum }w_{ij} \end{array}$$

where *k* represents the strength, and *w* represents the weight between the nodes *i* and *j*; in this case, *w*_*ij*_ was set as the correlation coefficient. This value represents a local connectivity of the node indicating how its connection with adjacent nodes is strong.

*Betweenness* represents how many times a node serves as a bridge between two other nodes; it is given by the formula:

$$\begin{array}{l} b_{i}=\underset{j<k}{\sum }\frac{p_{jk}\left(i\right)}{p_{jk}} \end{array}$$

where *p*_*jk*_ represents the number of shortest paths between nodes *j* and *k*, and *p*_*jk*_(*i*) is the number of shortest paths between *j* and *k* that pass through *i*. The higher this value is, the greater number of shortest paths passing through a node, indicating how that node is important in the average path between two nodes.

*Closeness* is the average length of the shortest path between a node and any other node; it can be considered as a measure of how long it will take to spread information to other nodes.

$$\begin{array}{l} L_{i}^{-1}=\frac{n-1}{\sum_{j\in Nj\neq i}d_{ij}} \end{array}$$

where *d* is the shortest path length between nodes *i* and *j*, and *n* is the number of nodes. The more central a node is, the lower its total distance is from all other nodes. It can be considered a measure of how information from a node reaches other nodes. Closeness differentiates with strength by the fact that it indicates how it relates to all other nodes in the network, not just the adjacent ones.

Betweenness, closeness, and strength represent centrality measures of the network that reveal the relative importance of nodes in the network, whether high centrality suggests to strongly affect other nodes due to their strong connection (28). Thus, centrality measures represent the degree in which a node influences other nodes in the network. However, even if they share this common feature, betweenness, closeness, and strength represent different forms of influence. We indicate that strength represents local connectivity as it measures its influence on the adjacent nodes. Betweenness indicates how that node is used to connect other nodes in the network, and finally, closeness represents how far a node is from all other nodes. This difference relies on how they are computed, whether betweenness is a measure that indicates how many times a node is located within the shortest path between two other nodes, strength is computed by examining how many connections are attached to each node, and closeness indicates how far a node is from all other nodes (29).

#### Network Analysis: Group Comparisons

In order to evaluate differences between ASD and TD networks, every network was non-parametric bootstrapped 1,000 times, obtaining for each bootstrap measures of strength, betweenness, and closeness of each node. Whereupon statistically significant differences were evaluated by the z test and adjusted by Bonferroni's correction (α = 0.05).

We used the *bootnet* package (30) from R (31) to perform the calculations.

#### Community Detection

Subsets of nodes that are densely connected to each other but less with others are referred to as communities (32). Modularity is a measure that indicates the quality of a particular division of the network (33). Modularity values range from −1 to 1, where a value of 0 indicates that the division is not better than random, while 1 theoretically indicates strong community structures; in practice, values for such networks fall between 0.3 and 0.7 (33).

There are different algorithms to detect graph communities; in our research, we used that proposed by Blondel et al. (34), which assigns a different community to each node of the network, then a node is moved to the community of its neighbors to achieve the highest positive contribution to modularity. This procedure is then repeated for all nodes until it obtains no further improvement. We used this method, as it has shown that it performs well in terms of accuracy (35). Communities were detected for each network using the *igraph* package (36).

## Results

### Comparison Between Groups

The *T*-test and Mann–Whitney test showed that children with ASD obtained lower scores in all components of the SIPI: interpretation (U = 2,083.50; *p* < 0.001), response evaluation [*t*_(155)_ = −3.01, *p* = 0.03], and response construction (U = 767.50; *p* < 0.001) compared to the TD children, with the exception of encoding (U = 2,414.50; *p* = 0.06). Regarding CST, the ASD group had lower scores in the emotions (U = 2,030.50; *p* < 0.001) and beliefs (U = 1,367.50; *p* < 0.001) components; there was no difference between the groups in the intentions component *(*U = 2,782.50; *p* = 0.26).

Finally, regarding ET, children with ASD showed difficulties in the negative mental states component [*t*_(155)_ = −2.97; *p* = 0.03]; however, there were no significant differences in the positive mental states component [*t*_(155)_ = −1.50; *p* = 0.13], the primary emotions component with negative valence [*t*_(155)_ = −0.18; *p* = 0.85], or the positive valence [*t*_(155)_ = 1.33; *p* = 0.18] compared to the TD group.

### Visualization of the Networks

**Figure 1:**
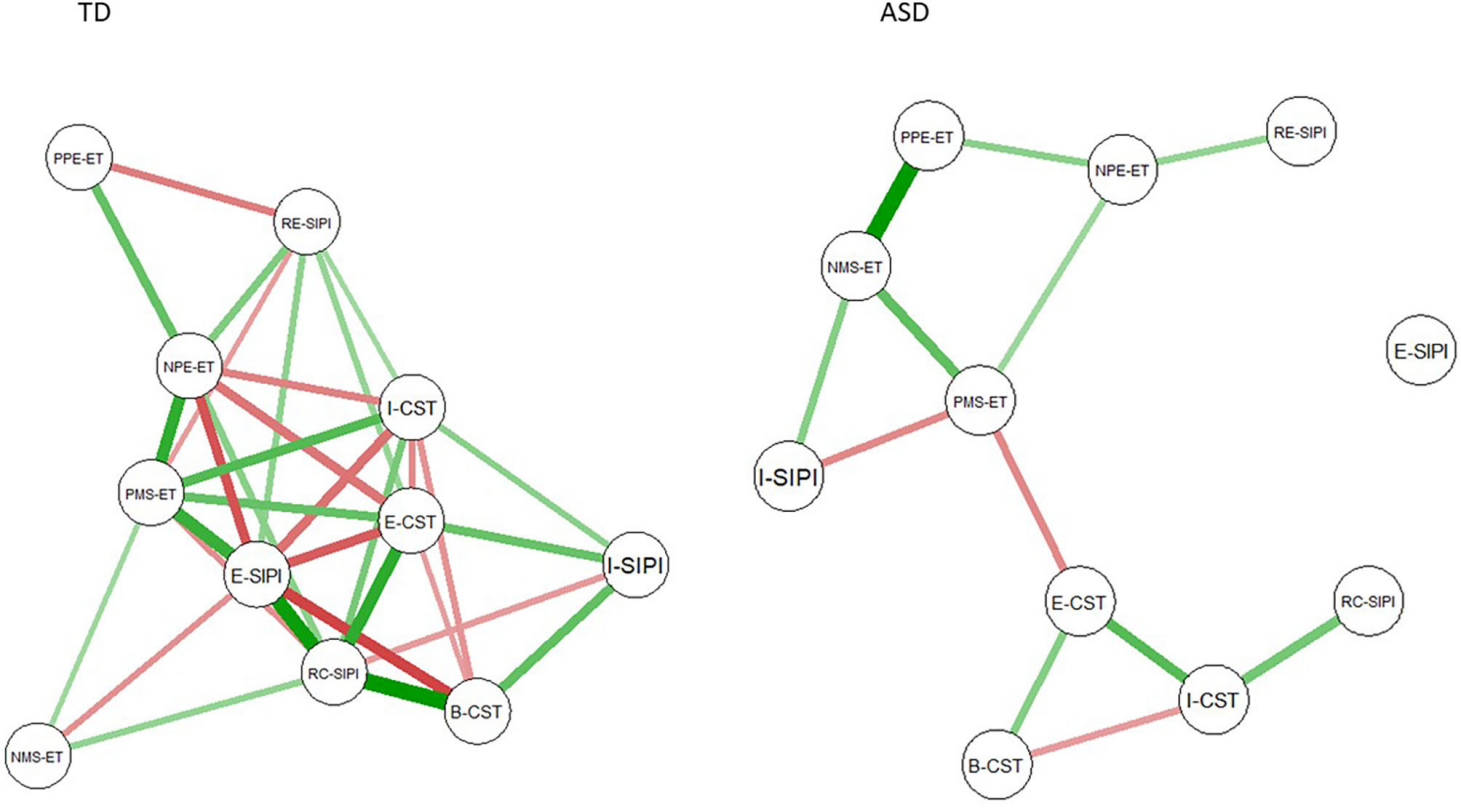
Graphs of typically developing (TD) and autism spectrum disorder (ASD) populations. Each node represents a social cognition (SC) domain. Strength is represented by the link's thickness, and closeness by the nodes' distance. Green and red links represent positive and negative correlation coefficients, respectively. B-CST, Belief-Comic Strip Task; E-CST, Emotions-Comic Strip Task; I-CST, Intentions-Comic Strip Task; NPE-ET, Negative primary emotions-Eyes Task; PPE-ET, Positive primary emotions-Eyes Task; NMS-ET, Negative mental states-Eyes Task; PMS-ET, Positive mental states-Eyes Task; E-SIPI, Encoding-Social information processing interview; I-SIPI, Interpretation-Social information processing interview; RC-SIPI, Response construction-Social information processing interview; RE-SIPI, Response evaluation-Social information processing interview.

Networks are represented in Figure 1, nodes represent SC components, lines between nodes represent correlations between measures, while widths of lines represent the strength of a correlation. Red and green lines represent positive (green) and negative (red) correlations. The node's distance represents closeness.

### Graph Measures

Graph analysis results are reported in Table 2 (which also contains the z tests performed). However, it was possible to evaluate significant differences between closeness and strength measures. Statistical analysis shows significant differences between the two networks regarding all the closeness and strength measures, where the graph of the TD sample reveals global greater values in each node for closeness and strength. Nodes in ASD's network showed greater betweenness values compared to nodes in TD's network except for NPE-ET and E-SIPI, which instead showed lower values compared to TD's nodes.

**Table 2:** Significant differences between groups (ASD and TD) for social cognition measures and canonical properties of graph analysis (betweenness, closeness, and strength) for each node (i.e., social cognition components).

**Social cognition measures**	**Measures score mean (SD)/ median [1st quartile−3rd quartile]**		**Betweenness mean (SD)**		**z**	**Closeness mean (SD)**		**z**	**Strength mean (SD)**		**z**
	**ASD**	**TD**	**ASD**	**TD**		**ASD**	**TD**		**ASD**	**TD**	
**COMIC STRIP TASK**											
Belief (B-CST)	1 [1–2]^*^	4 [2–4]^*^	4.33 (7.27)	2.28 (3.96)	−7.78^*^	0.01 (0.01)	0.04 (0.02)	27.95^*^	1.12 (0.72)	4.10 (2.80)	32.52^*^
Emotions (E-CST)	4 [4–4.75]^*^	5 [4–5]^*^	10.55 (10.55)	4.57 (6.03	−15.55^*^	0.01 (0.01)	0.04 (0.02)	28.48^*^	1.56 (0.78)	4.46 (2.71)	32.42^*^
Intentions (I-CST)	4 [3–5]	4 [3–5]	9.75 (10.90)	2.23 (4.48)	−20.19^*^	0.01 (0.01)	0.04 (0.02)	27.95^*^	1.26 (0.48)	4.16 (2.78)	32.43^*^
**EYES TASK**											
Negative primary emotions (NPE-ET)	12.11 (2.06)	12.16 (1.27)	4.88 (8.32)	8.43 (8.96)	31.26^*^	0.01 (0.01)	0.04 (0.02)	31.26^*^	0.98 (0.54)	4.27 (2.67)	38.08^*^
Positive primary emotions (PPE-ET)	11.52 (2.39)	11.08 (1.65)	9.77 (10.17	1.37 (3.84)	19.60^*^	0.01 (0.01)	0.03 (0.02)	19.60^*^	1.49 (0.66)	3.05 (3.01)	15.87^*^
Negative mental states (NMS-ET)	10.6 (2.56)^*^	11.64 (1.71)^*^	13.29 (10.28)	0.70 (2.50)	18.64^*^	0.01 (0.01)	0.03 (0.02)	18.64^*^	1.70 (0.67)	3.00 (3.13)	12.81^*^
Positive mental states (PMS-ET)	10.26 (2.40)	10.76 (1.69)	12.57 (12.65)	3.27 (5.07)	28.31^*^	0.01 (0.01)	0.04 (0.02)	28.31^*^	1.44 (0.63)	4.30 (2.69)	32.58^*^
**SOCIAL INFORMATION PROCESSING INTERVIEW**											
Encoding (E-SIPI)	4 [3–4]	4 [3–4]	3.99 (7.34)	6.94 (7.42)	8.94^*^	0.01 (0.01)	0.04 (0.02)	35.70^*^	0.86 (0.57)	4.84 (2.55)	48.03^*^
Interpretation (I-SIPI)	3 [2–3]^*^	3 [3–4]^*^	7.18 (9.65)	2.39 (4.89)	−13.99^*^	0.01 (0.01)	0.04 (0.02)	23.10^*^	1.36 (0.69)	3.45 (2.92)	22.10^*^
Response construction (RC-SIPI)	2 [1–3]^*^	4 [3–5]^*^	9.93 (10.71)	6.68 (7.12)	−7.98^*^	0.01 (0.01)	0.04 (0.02)	31.87^*^	1.38 (0.69)	4.83 (2.49)	42.14^*^
Response evaluation (RE-SIPI)	19.71 (2.15)^*^	29.73 (1.09)^*^	5.16 (7.92)	1.45 (3.48)	−13.57^*^	0.01 (0.01)	0.04 (0.02)	24.91^*^	0.95 (0.60)	3.43 (3.02)	25.43^*^

*ADS, autism spectrum disorder; TD, typically developing*.

* *p < 0.05*.

### Community Detection

**Figure 2:**
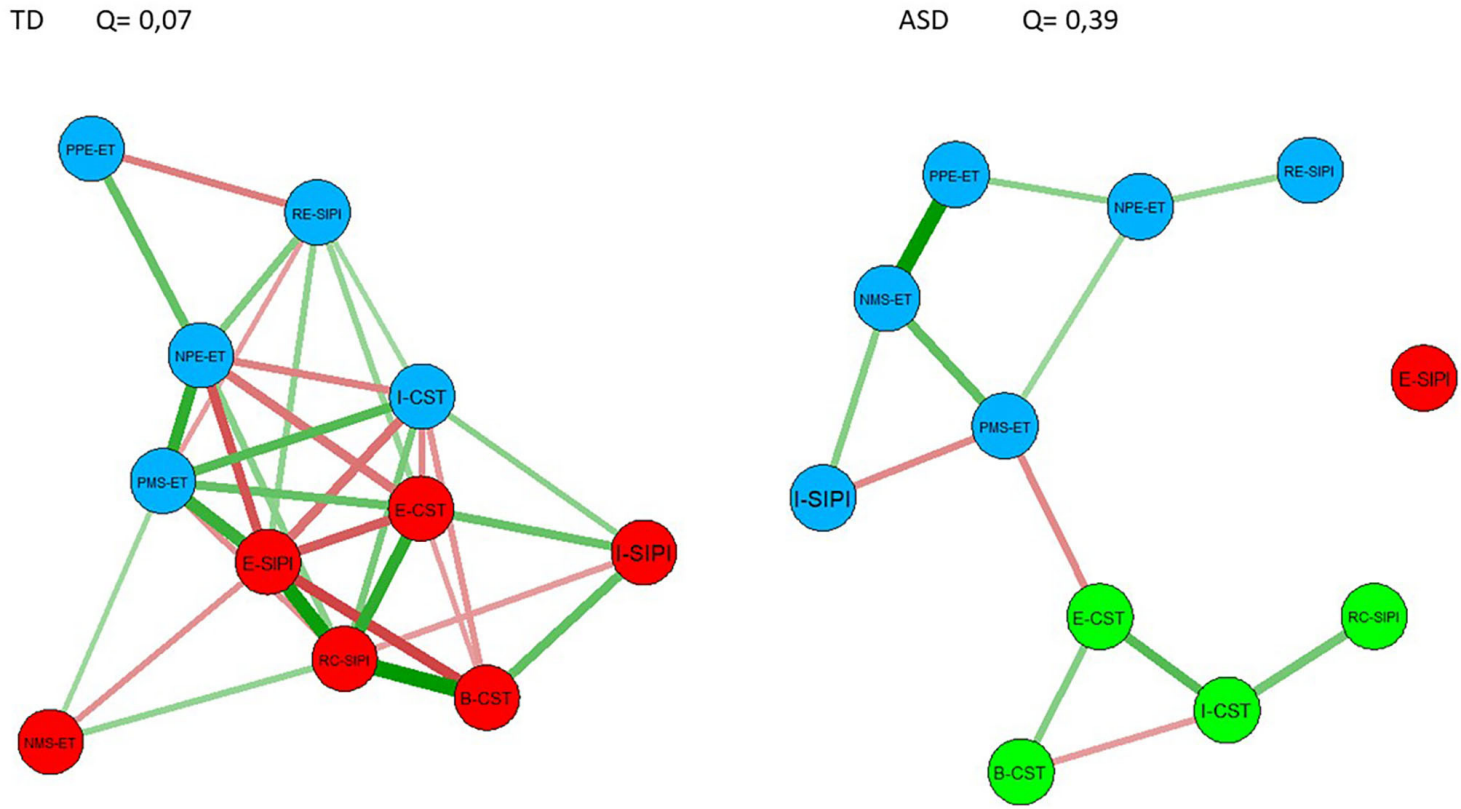
Subset of nodes found by community detection, nodes colored with the same color represent communities found for each network.

Communities detected are presented in Figure 2. Modularity estimated for the TD communities was low (Q = 0.07), indicating that the community's divisions were not better than random, suggesting that there are no communities in the network. In community detection for the ASD network though, we estimated a value of Q = 0.39, indicating that the network could be considered as divided into communities. ASD communities in the network are (a) I-SIPI, PMS-ET, NMS-ET, PPE-ET, NPE-ET, and RE-SIPI; (b) E-CST, B-CST, I-CST, and RC-SIPI; and (c) E-SIPI.

## Discussion

In the present study, we employed graph theory to map the structure of the SC network in children with ASD compared to TD children. Specifically, the aim was to build a network of SC domains/competencies to explore associations among interacting elements within this complex cognitive construct. Thus, we modeled the SC measures of children with ASD and TD as a complex system of interactions and further applied graph analysis to define important features therein for both groups.

Our results show that nodes in the ASD network show an overall higher betweenness compared to those in the TD network (except for the E-SIPI node and NPE-ET node, which showed a higher betweenness for the TD group). Nodes in the TD network showed an overall higher closeness and strength compared to the ASD network.

Community detection in the ASD network found three communities of nodes: the first community was composed of a single component, i.e., the E-SIPI node (Encoding) that is a community *per se*. This result suggests that people with ASD can encode social information, but this social information would not be utilized by the entire network. This is confirmed by the finding that this node in ASD showed lower scores in all the centrality measures compared to TD.

The second community was composed of the CST subdomains, namely, the E-CST node (Emotions), the B-CST node (Beliefs), and the I-CST (Intentions), to which is added an SIPI component: the RC-SIPI node (response construction), indicating that the construction of the social response is strictly related to the understanding of intentions, beliefs, and emotions. In the response construction, the child is asked: “to put himself/herself in the shoes” of the main character of the story and try to explain how he/she would act in the same situation (6). This ability is densely connected with the construction of a response in a social scenario.

The third community obtained was composed of domains as the ET such as NMS-ET (Negative Mental States), PMS-ET (Positive Mental States), PPE-ET (Positive Primary Emotions), NPE-ET (Negative Primary Emotions), and nodes representing domains evaluated by the SIPI names I-SIPI (Interpretation) and RE-SIPI (Response Evaluation). This community could indicate that the interpretation of the scene, as well as the assessment of whether the response given by the other is right or wrong, is closely related to the interpretation of information also of a visual type (interpretation of mental states based on visual cues). Specifically, the RE-SIPI component is crucial for processing social cues and subsequently social behavior, whereas I-SIPI component evaluates the tendency to attribute hostile intentions to other people in positive social situations and *vice versa*.

Furthermore, while the ET and CST are grouped in the same community, the components of the SIPI are divided among three communities, suggesting that the information required to complete this particular task must make more transitions between SC skills.

Indeed, *betweenness* represents the number of paths that pass through a node, higher betweenness in ASD, and the existence of communities could indicate that, to reach a determined node, information passes through and is influenced by other nodes before reaching it. Betweenness is a measure based on the concept of shortest path, which is the path between node A and node B with the minimum intermediary nodes. To clarify the results based on this concept, we can refer to the ASD network in Figure 1. If, for example, we take the PMS-ET and E-CST nodes, we can see that they form a “bridge” between two agglomerations of nodes, so regardless of the length of the shortest path, any path between the two agglomerates (which we have found are communities) will have to go through those nodes, resulting in a high betweenness for both. The same reasoning can be made for the other nodes of the two communities. E-SIPI node shows low betweenness since it is not connected in the network. The TD network has a more “balanced” situation, even though we have a betweenness that is not as wide as on most ASD nodes. Moreover, this kind of spread of information characterizes the communication between communities of nodes in ASD's network. The characteristic spread of information highlighted by our analysis could suggest that, within the SC network in ASD, a compensatory mechanism intervenes. However, this compensatory mechanism seems to become less efficient when one is facing a more complex social scenario, as the one presented in the SIPI (characterized by the simulation of a peer-to-peer social situation).

In a recent study, it was found that processing and understanding of complex social information measured through the SIPI could well-differentiate between children with ASD and TD rather than CST and ET (37). According to Pino et al. (3), intentions and emotion comprehension are lower-level competencies; these skills are learned earlier and are necessary for the construction of more complex abilities (6).

Our findings confirm that SIPI represents a complex task for ASD, thus the altered segregation and the altered use of information concerning this task may be related to such difficulties. SIPI is a complex task that is more similar to social context compared to the other tasks. Since it is based on the model of Crick and Dodge (15), planning a correct social response involves the use of several capacities (e.g., multiple evaluations, regulations, schemas, or rules). As seen in Figure 1, we found that the Encoding node is separated from the rest of the network, thus it presents lower centrality measures (strength, betweenness, and closeness) compared to TD. When a child is involved in a social situation, through encoding, he or she can give an interpretation of the social scene thus construct and make a response (15). Cooper et al. (38) have found a dissociation between encoding and recollection in people with ASD; our results support an impairment in the encoding process, which characterized people with ASD, which would consequently also involve other social skills. Specifically, our results suggest that individual with ASD seems to use other sources of information during the interpretation or the construction of a social response rather than a proper mechanism of encoding.

From community detection, we found that the TD network presents a network without communities, i.e., there are no subsets of nodes that are densely connected with each other; moreover, they present higher values of closeness (which indicates how information directly reaches other nodes) and strength (which represents how locally the connection is strong with adjacent nodes). Taken together, these results suggest that, in the TD network, communication between nodes is direct and has major weight compared to the ASD nodes communication. Moreover, as the TD network does not present multiple communities, the SC skills measured by the assessed tasks, only for TD, would compose a single domain, i.e., SC.

Even if these results must be interpreted with caution, it is important to know that the ASD network and the TD network fit the theoretical SC network proposed by Happé and Frith (9); further, our approach highlighted properties of the network that differentiate ASD and TD SC.

As recent literature has found that ASD is characterized by a delay of social abilities (3, 6) and VMA appears to be a good predictor of ToM ability (3, 18), our ASD sample was determined to have the same mental age as the TD group.

Our research confirms the results of Pino et al. (3) and Hobson (19), as matching group by VMA has yielded the same results in components of SC measured by test scores; specifically, they do not show significant differences in Intentions, as measured by the CST; in Primary Emotions with negative and positive valence, as measured by the ET; and Positive mental states. On the contrary, the two groups differ in negative mental state perception. The role of emotional valence in ASD is still being debated. Several studies suggest that the processing of negative emotions is most difficult for individuals with ASD (39–41). Ashwin et al. (42) consider the difficulty of processing negative emotions in ASD to be linked to an atypical function and structure of the amygdala. In their study, individuals with ASD were less accurate on the emotion recognition task compared to controls, but only for the negative basic emotions. According to Mazza et al. (40), emotional contagion for negative emotions of other people (like sadness, distress, suffering, anger) is important for adaptive social behavior. The lack of sharing experience when other people have negative emotions leads to a failure of appropriate empathic behavior in ASD people. In addition, the two groups showed differences in understanding emotions and beliefs, but not in the Intention component of the CST. We suggest that is consistent with the suggestion that the ability to understand others' beliefs and emotions is more complex with respect to the ability to understand the intentions of other people (3, 9). The latter ability occurs earlier in development as it is already present, to a certain extent, in TD infants (9). Thus, it is possible that in children with ASD, this ability becomes available later, and at the age of about 6 or 7 years, they gain the ability to understand the intentions of other people (3).

SC comprises various functions such as emotional processing, imitation, pretend play, ToM, empathy, and so on (9), but in this study, we consider only a part of this complex construct, and this can represent a limitation of our research. In the literature, at least part of the interest in ToM abilities is associated with the idea that people with ASD suffer from “mind-blindness” (3, 43). Social difficulty compromises interpersonal relationships and leads to social isolation in individuals with ASD. For this reason, it is important to study the SC network in the ASD group to create their SC profile and thereby understand how relations among its domains/components may affect their social behavior.

### Limitations

It is important to know that our research presents some considerable limitations. It must be pointed out that results must be interpreted with caution as our analysis describes connectivity between nodes/domains but did not give information with regard to how information is processed by nodes or the direction of the information flow that follows.

In fact, our results do not express how a node elaborates information in the network (e.g., filtering, selecting, select salient feature, etc.) that literature and future research could elaborate, although it is important to highlight that this approach clarifies important features of the network's connections. Another important limitation was that our samples were composed mostly of males. Moreover, our network is a simplified version of the Happé and Frith (9) SC network; thus, implementing other nodes in the model could lead to a better understanding of connections between SC domains.

### Future Directions

Future research could investigate specific contributions that SC domains provide in the network, deepening our understanding of under what processes social information passes between one domain and another. According to Vagnetti et al. (44), future research should be directed toward understanding how node processes effectively contribute to the network (e.g., by integrating or controlling information) and how edges change over time. Indeed, these structures could be time-dependent, especially during early development, and it would be interesting to understand when the structure of SC becomes fixed. In addition, it would also be interesting to investigate how intervention in ASD could change the network, e.g., connections and/or their weight, and it would be interesting to know if a variant version of functioning could mitigate social difficulties associated with ASD. It would be further interesting to consider a longitudinal study that would explore if and how the SC network structure becomes time-dependent.

We support the idea that a rehabilitation intervention could try to work on the strength of the edges to improve the network connections.

Our results showed that ASD domains of the SC network were isolated compared to TD, which were connected. The TD network reflects the theoretical SC network model proposed by Happé and Frith (9), where components are connected to each other.

Network indices comparison between the two groups shows a different communication role and statistical weight of the SC network nodes. These differences deserve a better understanding because they could provide an explanation of social functioning difficulties in ASD as well as their social isolation.

## Conclusion

In conclusion, our research has found a connected SC network in children with ASD and TD, although important differences emerged in nodal communication—a phenomenon possibly related to the SC deficits presented by ASD.

While the TD SC network is characterized by a single domain, we found that ASD is characterized by node communities, thus higher betweenness for ASD nodes reflects the communication between communities, while higher strength and closeness for the TD highlight important contributions of each node in the network. SIPI represents a key point in understanding differences between groups. Our findings suggest that, for ASD, social information encoding performs a reduced contribution in the network; moreover, some types of information (measured by CST and ET) seem more relevant depending on the process involved (Response construction or Interpretation and Response evaluation).

## Data Availability Statement

The raw data supporting the conclusions of this article will be made available by the authors, without undue reservation.

## Ethics Statement

The studies involving human participants were reviewed and approved by ASL L'AQUILA—PROTOCOL NUMBER 186061/17. Written informed consent to participate in this study was provided by the participants' legal guardian/next of kin.

## Informed Consent

Informed consent was obtained from parents and oral assent from children in order to collect demographic and score variables.

## Author Contributions

All authors listed have made a substantial, direct and intellectual contribution to the work, and approved it for publication.

## Conflict of Interest

The authors declare that the research was conducted in the absence of any commercial or financial relationships that could be construed as a potential conflict of interest.
